# Evidence for ice-ocean albedo feedback in the Arctic Ocean shifting to a seasonal ice zone

**DOI:** 10.1038/s41598-017-08467-z

**Published:** 2017-08-15

**Authors:** Haruhiko Kashiwase, Kay I. Ohshima, Sohey Nihashi, Hajo Eicken

**Affiliations:** 1 National Institute of Polar Research, Tachikawa, 190-8518 Japan; 2 Institute of Low Temperature Science, Hokkaido University, Sapporo, 060-0819 Japan; 3 Arctic Research Center, Hokkaido University, Sapporo, 001-0021 Japan; 4 National Institute of Technology, Tomakomai College, Tomakomai, 059-1275 Japan; 5 International Arctic Research Center, University of Alaska Fairbanks, Fairbanks, Alaska 99775-7340 USA

## Abstract

Ice-albedo feedback due to the albedo contrast between water and ice is a major factor in seasonal sea ice retreat, and has received increasing attention with the Arctic Ocean shifting to a seasonal ice cover. However, quantitative evaluation of such feedbacks is still insufficient. Here we provide quantitative evidence that heat input through the open water fraction is the primary driver of seasonal and interannual variations in Arctic sea ice retreat. Analyses of satellite data (1979–2014) and a simplified ice-upper ocean coupled model reveal that divergent ice motion in the early melt season triggers large-scale feedback which subsequently amplifies summer sea ice anomalies. The magnitude of divergence controlling the feedback has doubled since 2000 due to a more mobile ice cover, which can partly explain the recent drastic ice reduction in the Arctic Ocean.

## Introduction

Ice-albedo feedback is a key aspect of global climate change. In the polar region, a decrease of snow and ice area results in a decrease of surface albedo, and the intensified solar heating further decreases the snow and ice area^1^. In the Arctic Ocean, recent observations have revealed major reductions in summer ice extent^2, 3^, thinning of sea ice^4, 5^, and a shift from perennial to seasonal sea ice^6–8^, particularly after the 2000s. It is well established that climate change signals are amplified in the Arctic^9–11^ and that such “polar amplification” is associated with ice albedo feedbacks^12–14^.

Until recently, the Arctic Ocean has been characterized by a thick multiyear ice cover that persisted throughout the summer, with melt confined to its upper surface^15^. In the seasonal ice zone, presence of an open water fraction with a much lower albedo results in high solar radiation absorption by the upper ocean^16, 17^, which in turn serves as the dominant heat source for sea ice lateral and bottom melt^18, 19^. Since the seasonal ice zone is dominated by thin and undeformed first-year ice, the melting of sea ice immediately increases the fraction of open water in the ice-covered area and thus drives up absorption of solar energy in the upper ocean. Hence, in regions dominated by seasonal ice such as the Southern Ocean and the Sea of Okhotsk, ice-albedo feedback due to the albedo contrast between water and ice surfaces, termed ice-ocean albedo feedback, enhances summer sea ice retreat^20^ and partly controls interannual variability of the ice cover^21, 22^.

Recently, such feedback effects have also received attention in the context of drastic reductions in summer Arctic sea ice extent^19, 23–26^ and the shift from perennial to seasonal sea ice. Satellite observations indicate a significant positive trend in solar heating of the upper ocean associated with recent changes in sea ice concentration and/or increase in ice-free area^19^. However, key questions, such as how much of the variation in sea ice retreat and the recent sea ice reduction are explained by heat input through the open water fraction, or the specific physical processes at work in triggering and translating the feedback, remain unanswered.

**Figure 1:**
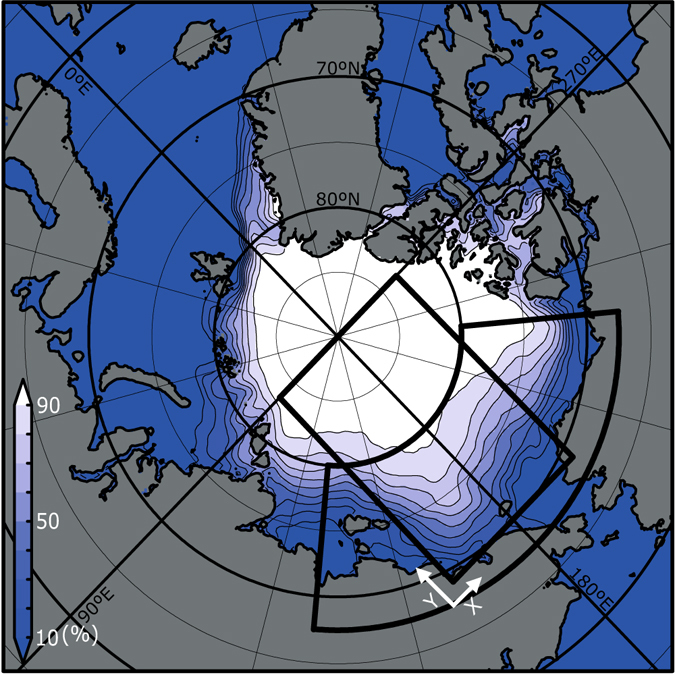
Map of the Arctic Ocean with September sea ice concentration averaged from 1979 to 2014. The heat budget analysis and calculation of ice divergence were made for the fan-shaped area. The simplified model was applied for the rectangular area. The map is drawn by GrADS 2.0.2 (available from http://cola.gmu.edu/grads/grads.php).

Here we show the dominance of heat input through the open water fraction on sea ice loss and its variation, which is a necessary condition for ice-ocean albedo feedback, based on the relationship between sea ice retreat and heat budget over the ice-covered area. Then we explore the specific trigger of the feedback effect, and examine whether ice melt is in fact amplified significantly by this feedback, and whether the drastic reduction in summer ice extent can be explained by this feedback, based on the combined analysis of satellite observations and a simplified ice-ocean coupled model. We selected the Pacific Arctic Sector (fan-shaped area in Fig. 1) as the main study area. This region experienced the largest reductions in summer ice extent and volume anywhere in the Arctic Ocean beginning in the 2000s. Interannual variation of ice retreat in this region explains about 86% of the variance over the entire Arctic Ocean (*n* = 36, *p* < 0.001; Supplementary Fig. S1).

## Results

### Dominance of heat input through the open water fraction

**Figure 2:**
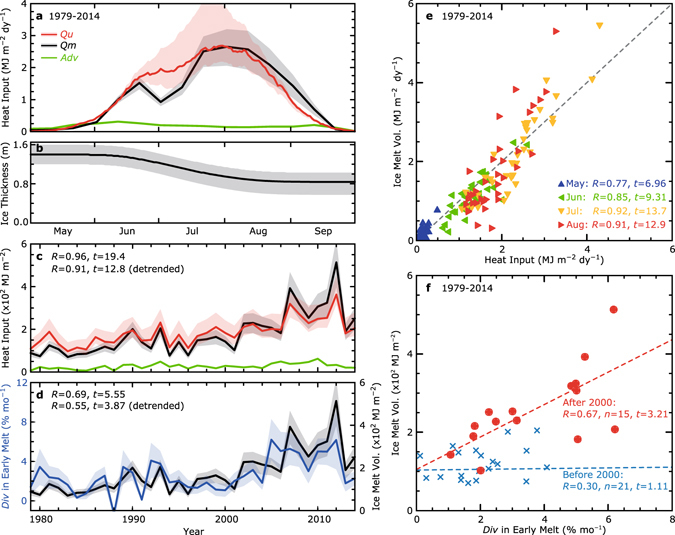
Results of heat and sea ice budget analyses. Seasonal evolutions of (**a**) heat input through the open water fraction (*Qu*, red line), ice melt volume (*Qm*, black line), and the volume of ice export (*Adv*, green line), and (**b**) mean ice thickness calculated from the surface melt by heat input at the ice surface. The volume of ice is converted to the heat required for the equivalent amount of ice melt. (**c**) Interannual variations in *Qu*, *Qm*, and *Adv* accumulated from May to August. (**d**) Interannual variations of ice divergence (*Div*) averaged from mid-May to early-June (blue line) and yearly *Qm*. (**e**) Scatter plot of *Qu* and *Qm*. Monthly means (May to August) for each year are plotted. (**f**) Scatter plot of *Div* averaged from mid-May to early-June versus yearly *Qm*. Blue crosses and red circles indicate values before and after 2000, respectively. Dashed lines indicate the regression line for both periods. Uncertainties due to errors in the satellite observations are shown as shaded envelopes (see Methods).

For the ice-covered area defined by ice concentrations >15%, we have analyzed the daily heat budget separately for the water and ice surfaces from 1979 to 2014 (see Methods). During the summer season, net heat flux at the water surface (*Fw*) is much larger than that at the ice surface (*Fi*), because shortwave radiation is the dominant component of heat budget in the analysis area (Supplementary Fig. S2). Here we focus on the amount of heat input into the upper ocean through the open water fraction (*Qu*), and compare this heat with the volume of sea ice melt (*Qm*) which was calculated from the observed decrease of ice area multiplied by mean ice thickness, accounting also for the decrease by ice advection (*Adv*) (Fig. 2). In the calculation of *Qu*, the analysis area varies as the ice retreats. This implicitly assumes that heat exchange between ice pixels and open ocean pixels outside of the ice edge is negligible. Since the ice migration scale of 20–30 km under typical atmospheric synoptic processes in the sea ice zone^27^ is much smaller than the scale of the entire analysis area, this assumption is valid for a zeroth-order approximation. It should be noted that, since most of the sea ice area exceeds 80% ice concentration even in summer, the amount of heat input at the ice surface (*Qi*) is comparable to *Qu* and thus contributes significantly to ice melt. In this paper, we assume that heat input at the sea ice surface resulting in surface melt is exclusively used to reduce ice thickness. We calculated the seasonal evolution of mean ice thickness which decreases from the initial thickness of 1.4 m, as observed by ICESat (Supplementary Fig. S3), to 0.86 m through surface melt, based on the climatological mean of heat budget calculation (Fig. 2b). Then, we used this for the calculations of *Qm* and *Adv* (see Methods). While this simplification is a zeroth-order approximation, it is valid at least for examining interannual variability, considering that the relative standard deviation of yearly *Qi* (6%) is much smaller than that of yearly *Qu* (32%).

Estimates from the heat budget analysis and satellite observations show that *Qu* corresponds well quantitatively with *Qm* both for seasonal and interannual variations (Fig. 2a,c and e). Correlation coefficients between *Qu* and *Qm* are statistically significant (*n* = 36, *p* < 0.001) as 0.77, 0.85, 0.92, and 0.91 for the monthly mean from May to August, respectively (Fig. 2e). The correlation coefficient between the yearly values is also statistically significant as 0.96, and 0.91 for detrended variations (*n* = 36, *p* < 0.001; Fig. 2c). However, results of the heat budget analysis have a relatively large uncertainty mainly due to the formation of melt ponds^28^. In this paper, we have estimated the fraction of melt ponds from the temporal change in sea ice albedo, and then assessed the influence of such effect (see Methods). The error in *Qu* caused by melt ponds is shown by red shading in Fig. 2. The error in *Qm* mostly due to the uncertainty of mean ice thickness is also indicated by gray shadings. It is noted that ice export from the fan-shaped area and its interannual variation (green lines in Fig. 2a and c) are much smaller than *Qu* and *Qm*. These results indicate that ice retreat in the Pacific Arctic is mainly explained by the ice melt due to heat input through the open water fraction, implying that the necessary condition for ice-ocean albedo feedback is satisfied in the study area.

### Relationship between divergent ice motion and subsequent ice melt

Previous studies in the Antarctic^21^ and the Sea of Okhotsk^22^ pointed to the importance of divergent ice motion caused by offshore-ward winds in the early melt season for subsequent ice retreat. Unlike these oceans, the divergence in ice motion of the Pacific Arctic is determined mainly by the Transpolar Drift, the Beaufort Gyre, and the migration of the ice edge. To deal with these factors simultaneously, we have calculated the mean ice divergence over the ice-covered area (*Div*) using the ice drift velocity derived from satellite observations (see Methods). Comparisons between *Div* and ice retreat conditions (Supplementary Fig. S4) show that *Div* during the melt season significantly correlates with the simultaneous/subsequent ice concentration and ice melt volume. Particularly, *Div* in the earliest stage of the melt season (from mid-May to early-June) has the highest correlation with the sea ice retreat lagged by 1–2 months, with high correlation persisting through the end of August. Thus, this early *Div* is also well correlated with the yearly value of *Qm* (Fig. 2d), with a correlation coefficient of 0.69, and 0.55 for detrended variations (*n* = 36, *p* < 0.001). These suggest that the divergent ice motion in the early melt season can be a trigger of ice melt acceleration through ice-ocean albedo feedback. After the 2000s, such relationship has likely become stronger, suggested by a much higher regression coefficient than that prior to 2000 (Fig. 2f).

### Representation of feedbacks through a simplified model

**Figure 3:**
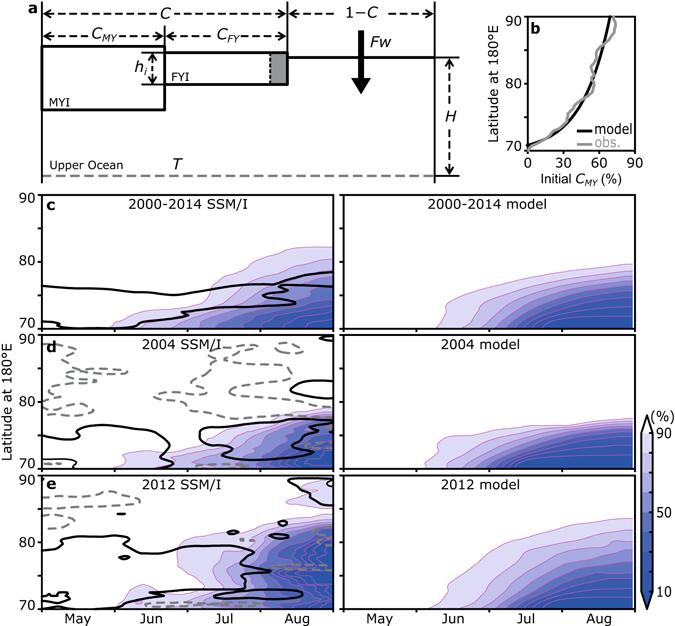
Results of the simplified model. (**a**) Schematics of the model. (**b**) Latitudinal distribution of multiyear ice (*C*_*MY*_) from satellite observations (gray line) and as used in the model (black line). (**c**–**e**) Meridional-time evolution of sea ice concentration obtained from satellite observations (left panels) and from the simplified model (right panels). Thick contours in left panels delineate cases when absolute sea ice divergence (solid black line) and convergence (dashed gray line) exceed 3% mo^−1^. Figures are drawn by GrADS 2.0.2 (available from http://cola.gmu.edu/grads/grads.php).

Focusing on the period after 2000, we have examined the effect of ice-ocean albedo feedback on the summer retreat of Arctic sea ice cover by using a simplified ice-upper ocean coupled model^27^. The model is based on assumptions similar to those in the heat budget analysis: only heat input through the open water fraction, *Fw*, is used for ice area decrease through melt (Fig. 3a), and heat input at the top of the ice surface is only used for a reduction in mean ice thickness. We use the seasonally changed ice thickness of Fig. 2b. The upper ocean is represented by a mixed layer of thickness *H* with a uniform temperature *T*. Exchanges of heat and water with the ocean below the mixed layer and the surrounding grid cells are assumed to be zero. The sea ice area *C* is divided into first- and multiyear ice (*C*_*FY*_ and *C*_*MY*_), respectively (*C* = *C*_*FY*_ + *C*_*MY*_). Here we assume that the melt of first-year ice and that of multiyear ice are proportional to their respective areal fraction. However, only first-year ice can be reduced in area through melt, not multiyear ice. The local heat balance of the upper ocean is given by

1 $${c}_{w}{\rho }_{w}H\frac{dT}{dt}=Fw(1-C)+{L}_{f}{\rho }_{i}{h}_{i}\frac{C}{{C}_{FY}}\frac{d{C}_{FY}}{dt},$$

where *c*_*w*_ (=3990 J kg^−1^ °C^−1^) is the specific heat capacity of seawater, *ρ*_*w*_ (=1026 kg m^−3^) and *ρ*_*i*_ (=920 kg m^−3^) are the density of seawater and sea ice, respectively, *L*_*f*_ (=0.276 MJ kg^−1^) is the latent heat of fusion for sea ice, and *h*_*i*_ is the mean ice thickness. The melt rate of first-year ice is parameterized as

2 $$-{L}_{f}{\rho }_{i}{h}_{i}\frac{d{C}_{FY}}{dt}={c}_{w}{\rho }_{w}{K}_{b}{C}_{FY}(T-{T}_{f}),$$

where *K*_*b*_ (=1.2 × 10^−4^ m s^−1^) is the bulk heat transfer coefficient between ice and ocean^27^, and *T*_*f*_ (=−1.86 °C) is the freezing temperature. Equations (1) and (2) can be combined and rewritten as,

3 $$H\frac{dT}{dt}=\frac{Fw(1-C)}{{c}_{w}{\rho }_{w}}-{K}_{b}C(T-{T}_{f}).$$

Then, the model is extended two-dimensionally, with effects of ice motion, advection, diffusion, and mechanical redistribution terms introduced; equation (2) is modified as follows,

4 $$\frac{dC}{dt}=-\frac{{c}_{w}{\rho }_{w}{K}_{b}(T-{T}_{f})}{{L}_{f}{\rho }_{i}{h}_{i}}{C}_{FY}-u\frac{\partial C}{\partial y}+{A}_{H}\frac{{\partial }^{2}C}{\partial {y}^{2}}+{\psi }_{C},$$

where *u* is the ice velocity. The spatial scale of grid cells is set to 28 km, and the lateral diffusion coefficient *A*_*H*_ is set to 5.0 × 10^3^ m^2^ s^−1^, based on a previous study^27^. The term *ψ*_*C*_ represents ice resistance, which redistributes the ice concentration so as not to exceed 99%.

Here we simulate the meridional time evolution of ice retreat in the rectangular area in Fig. 1. During the actual ice retreat, heat input through the open water fraction results in bottom and lateral melt, and deterioration and fragmentation of ice floes^29^ also have a significant impact through disproportionately greater lateral melt for smaller floes^30^. Since a significant portion of first-year ice (and brash ice) eventually melts completely and hence bottom melt contributes indirectly to decreases in sea ice area in addition to lateral melt, the simplified model considers these processes in a bulk fashion. In all the model runs, the initial ice concentration is set to 99%, the initial distribution of multiyear ice (Fig. 3b) is based on satellite observations, and net heat input at the water surface (Supplementary Fig. S5a) is obtained from the heat budget calculation.

The ice retreat averaged over 2000–2014 is successfully reproduced by the model using the ice drift velocity averaged over the same period (Fig. 3c, referred as the basic run). When compared with a simulation for which ice motion has been excluded (Supplementary Fig. S5b and c), the accumulated ice melt at the end of August in the basic run is enhanced 2.0 times, even though ice motion changes the ice concentration only by a few percent directly. These results illustrate how enhanced ice melt is triggered by divergent ice motion, such that ice-ocean albedo feedback can partly control the seasonal evolution of ice retreat in the Arctic Ocean. The model also points to the distribution of multiyear ice as another key factor constraining ice retreat in the Arctic Ocean (Supplementary Fig. S5d). Then, we consider the inherent time scale of this coupled system. Equations (2) and (3) are combined as,

5 $${L}_{f}{\rho }_{i}{h}_{i}\frac{d(1-C)}{dt}=Fw(1-C)-{c}_{w}{\rho }_{w}[\frac{dT}{dt}+{C}_{MY}(T-{T}_{f})].$$

If we assume constant values of *Fw* and *h*_*i*_ for simplicity, we can solve this equation for 1 − *C* as

6 $$1-C=\beta \,\exp \,[\frac{Fw}{{L}_{f}{\rho }_{i}{h}_{i}}t]+\gamma (t),$$

where *β* is a constant, and *γ*(*t*) is the influence of heat stored in the upper ocean. We obtain an e-folding time for this solution of *L*_*f*_ *ρ*_*i*_ *h*_*i*_/*Fw*, which serves as a measure of the inherent time scale of this coupled system. The estimated time scale of ~1.4 months during the early melt season (assuming constant values of *Fw* = 100 W m^−2^ and *h*_*i*_ = 1.4 m) explains the suggested lag time of enhanced melt relative to the trigger of ice divergence (Supplementary Fig. S4).

Ice retreat in extreme years such as 2004 and 2012, with weak and strong ice divergence, respectively (Fig. 2d), are also reproduced well by the model. Experiments are conducted under the same conditions as those of the basic run except for ice motion; results consistent with satellite observations (Fig. 3d and e) point to the key role of ice divergence in the context of feedback processes. Because ice-ocean albedo feedback sensitivity has increased beginning in the 2000s (Fig. 2f), a slight difference in ice motion can cause substantial deviations from climatological ice retreat through the amplifying effect of this feedback. These results also suggest that ice motion in the early melt season may possess predictive skill in seasonal sea ice forecasts in this sector of the Arctic Ocean.

## Discussion

**Figure 4:**
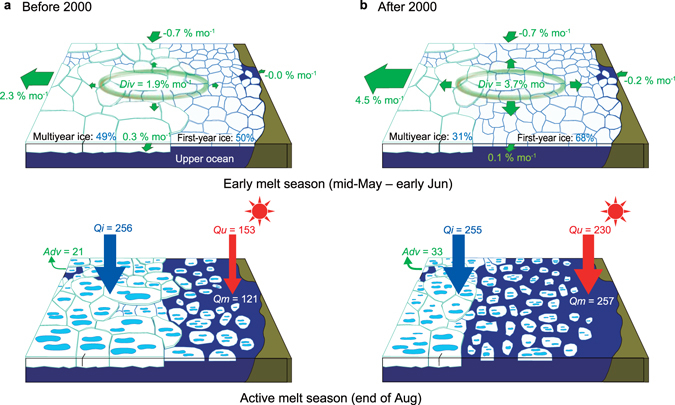
Schematic of ice and heat budgets during seasonal ice retreat. Mean ice conditions and heat budgets for the two periods (**a**) 1979–1999 and (**b**) 2000–2014 are shown. Divergent ice motion in the early melt season induces a small reduction in ice concentration (upper panel). A key finding is that although the direct contribution of doubled divergent ice motion after 2000 to the ice concentration reduction is small, this trigger accelerates ice melt through the enhanced solar heat input over the open water fraction (ice-ocean albedo feedback) until the end of August (lower panel). All values in the lower panel are ones accumulated from May to August and standardized as heat per unit area (MJ m^−2^), and the volume of ice is converted to the equivalent heat required for ice melt.

Findings from this study show that the feedback effect triggered by early-season divergent ice motion plays a key role in the seasonal evolution and interannual variation of sea ice retreat in the Pacific Arctic, particularly since the early 2000s. Below, we consider the contribution of such feedback to recent reductions in ice extent and volume, based on a comparison between mean states before and after 2000 (Fig. 4). In the early melt season, sea ice concentration sustains nearly 100% for both time periods, while, the fraction of multiyear ice based on ice age data^31^ has decreased from 49 to 31%. This reduction affects sea ice dynamics, in particular through decreases in ice mechanical strength and internal ice interaction forces, and increases in ice deformation rates^32^. These outcomes in turn increase the momentum flux from the atmosphere to the ocean^33, 34^, and strengthen anticyclonic circulation in the Beaufort Gyre with a steepening sea surface height anomaly after the early 2000s^35^. As a result of such changes, ice drift speed has significantly increased^36, 37^, likely responsible for the increase in early summer *Div* from 1.9 to 3.7% mo^−1^. Although the direct contribution of the increased divergence to reductions in ice concentration is quite small, accumulated heat absorption by the upper ocean through the end of August has gradually increased through ice-ocean albedo feedback, with an increase by a factor of up to 1.5 (from 153 MJ m^−2^ to 230 MJ m^−2^). This increased heat uptake can explain about 70% of the observed 2.1-fold increase in total sea ice melt (from 121 MJ m^−2^ to 257 MJ m^−2^). This contrast in the increase in annual ice melt compared to heat input is also evident in Fig. 2d, and is partly explained by the continuing decline in mean ice thickness^4, 5^.

Other factors such as changes in atmospheric circulation patterns^38^, influence of cloud cover^39, 40^, longwave radiative forcing due to anthropogenic CO_2_ emission^41^, melt pond distribution in the early summer season^42^, release of the solar heat stored in a near-surface layer of the ocean^43^, and increases in the heat inflow through Bering Strait^44^ may also contribute to drastic ice reductions. However, we note that these factors are intrinsically linked to divergence in the ice pack, because increased heat input from any source may enhance sea ice mobility. Thus, this study provides a new perspective on the observed drastic ice reduction in demonstrating, through modeling and analysis of remote sensing data, that ice divergence in the early melt season is a key trigger for amplification of ice retreat through ice-ocean albedo feedback. This finding also suggests that early-season ice divergence is associated with a substantial skill for seasonal ice prediction. A detailed analysis for the impact of individual processes on sea ice retreat at the local scale is beyond the scope of this study. Future work will require comprehensive analysis of output from fully coupled climate models along with time series of relevant sea ice quantities.

## Methods

### Data

In this study, daily sea ice data are derived from the Nimbus 7 Scanning Multichannel Microwave Radiometer (SMMR) and the Defense Meteorological Satellite Program (DMSP) Special Sensor Microwave Imager (SSM/I and SSMIS) from 1979 to 2014. The vertical and horizontal brightness temperatures^45, 46^ are provided by National Snow and Ice Data Center (NSIDC) and sea ice concentration is derived using the Bootstrap Algorithm^47^. For sea ice drift velocity, we use the dataset^48^ derived from a wide variety of sensors such as AMSR-E, SSM/I, AVHRR, IABP Buoys, and etc., provided by NSIDC. Spatial distribution of multiyear ice is estimated using the ice age data which is based on the particle tracking method^31^. Air temperatures and dew point temperatures at 2 m, wind speed at 10 m, and total cloud cover are obtained from the ECMWF Interim Re-analysis (ERA-Interim^49^), and then interpolated onto the 25 km polar stereographic grid with a Gaussian weighting function. For significance test of the statistics in this paper, we have performed the two-tailed t-test throughout the paper. Here *n* and *p* indicate sample number and significance probability, respectively.

### Heat budget analysis

Using sea ice observation data and meteorological reanalysis data, we have calculated the net heat budget at the water and ice surfaces which is expressed as the sum of shortwave and longwave radiation, sensible and latent heat fluxes^50^, over the ice-covered area of the Pacific Arctic (Supplementary Fig. S2). Then, the amount of heat input into the upper ocean through the open water fraction per unit area (*Qu*) is calculated from

7 $$Qu=\frac{\sum _{k}[F{w}_{k}(1-{C}_{k})S{g}_{k}]}{Se},$$

where *Fw* is the net heat budget at the water surface, *C* is the ice concentration, *Sg* is a unit grid cell area (≈25 km × 25 km), with subscript *k* denoting all sea ice pixels with *Fw* > 0 W m^−2^ in the analysis area, and *Se* (=Σ_*k*_ *Sg*_*k*_) as the extent of sea ice cover. Similarly, heat input at the ice surface and melt ponds are calculated as,

8 $$Qi=\frac{\sum _{k}[F{i}_{k}(1-f{p}_{k}){C}_{k}S{g}_{k}]}{Se},$$

9 $$Qp=\frac{\sum _{k}[F{p}_{k}f{p}_{k}{C}_{k}S{g}_{k}]}{Se},$$

where *Fi* and *Fp* are the net heat fluxes at the ice and melt pond surfaces, respectively, and *fp* is the areal fraction of melt ponds on sea ice. Detailed procedures for estimations of ice albedo and *fp* are described in the next section.

### Estimation of ice surface albedo and fraction of melt ponds

Since the distribution of melt ponds, a key factor in the heat budget of ice-covered area, is difficult to derive from the satellite observation directly, we have estimated the time evolution of melt pond fraction from a combination of several empirical approaches. First we estimated the ice albedo (*α*_*i*_) which varies in response to sea-ice surface conditions. Based on data for ice age^31^, we classified sea ice as first- and multiyear ice and then parameterized their albedos separately^51, 52^. Surface albedos for each ice type are determined from the number of days elapsed since onset of melt as derived from satellite microwave radiometer data^53^. Supplementary Fig. S6 shows a comparison of mean surface albedo calculated from the combination of this ice surface albedo and the ice concentration (i.e., *α* = *α*_*i*_ *C* + *α*_*w*_ (1 − *C*)) with that derived directly from AVHRR satellite observations^54^. These two independently derived albedos show similar seasonal evolutions, validating this approach. Then, we estimated the fraction of melt ponds (*fp*) as a function of *α*_*i*_, based on *in situ* observations^52, 55^ as follows:

10 $$f{p}_{FY}=\frac{0.53-{\alpha }_{FY}}{0.34},$$

11 $$f{p}_{MY}=\frac{0.65-{\alpha }_{MY}}{0.45},$$

where subscripts *FY* and *MY* denote the first- and multiyear ice, respectively.

### Estimation of ice melt volume and divergence

The volume of ice melt and ice divergence are estimated from ice concentration and ice drift data as schematically shown in Supplementary Fig. S7. We estimate the volume of ice loss (Δ*Vi*) and ice export from the boundary of analysis area *B* (Δ*Vo*) during the time step Δ*t* as follows,

12 $${\rm{\Delta }}Vi={h}_{i}[\sum _{k}{({C}_{k}S{g}_{k})}_{t=t1}\,-\,\sum _{k}{({C}_{k}S{g}_{k})}_{t=t1-{\rm{\Delta }}t}],$$

13 $${\rm{\Delta }}Vo={h}_{i}{\int }_{B}(C{\bar{u}}_{o}{\rm{\Delta }}t)dl,$$

where *h*_*i*_ is the mean ice thickness in the analysis area, and ${\bar{u}}_{o}$ is the outward component of sea ice drift normal to the boundary averaged over Δ*t*. To reduce the influence of errors in sea ice concentration data, we set Δ*t* as 10 days. Among the sea ice properties, ice thickness is by far the least observed. Here we calculate the reduction of ice thickness due to surface melt. According to the comparison of ice mass balance measurements with heat budget analysis^56^, ~40% of the heat absorbed into the ice surface contributes to the reduction of ice thickness. Based on this work, we can obtain a time-dependent ice thickness as,

14 $${h}_{i}(t)={h}_{0}-0.4\frac{{\int }_{t}\overline{Fi\,}dt}{{L}_{f}{\rho }_{i}},$$

where *h*_0_ is the initial ice thickness, $\overline{Fi}$ (=${{\rm{\Sigma }}}_{k}(F{i}_{k}S{g}_{k})/Se$) is the mean heat input at the ice surface obtained from the heat budget analysis, *L*_*f*_ (=0.276 MJ kg^−1^) is the latent heat of fusion for sea ice with salinity of 6 psu^57^, and *ρ*_*i*_ (=920 kg m^−3^) is the density of sea ice. We use an initial ice thickness of 1.4 m, corresponding to the mean ice thickness in the analysis area in the spring season (February through May), obtained from ICESat observations^58^ from 2003 through 2008 (Supplementary Fig. S3). Since ice loss is caused only by ice melt and ice export, the volume of sea ice melt per unit area (Δ*Vm*) during Δ*t* is obtained from

15 $${\rm{\Delta }}Vm=\frac{{\rm{\Delta }}Vi-{\rm{\Delta }}Vo}{Se}.$$

Then the volume of sea ice melt per day is converted to the corresponding heat according to

16 $$Qm=\frac{{L}_{f}{\rho }_{i}{\rm{\Delta }}Vm}{{\rm{\Delta }}t}.$$

The ice divergence (*Div*) during Δ*t* is estimated as the ice area export from the boundary of analysis area *B* and ice edge *E* (defined as the 15% ice concentration contour), standardized by the sea ice extent *Se*;

17 $$Div=\frac{{\int }_{B}(C{\bar{u}}_{o}{\rm{\Delta }}t)dl+{\int }_{E}(C{\bar{u}}_{o}{\rm{\Delta }}t)dl}{Se}.$$

### Error analysis

Sea ice quantities obtained from satellite observations are subject to varying levels of error. Here, we assess the uncertainty of results by considering the error for key variables, *Qu*, *Qm*, and *Div*, derived from satellite observations.

*Qu* is obtained as the product of open water fraction and net heat flux at the water surface. Since the shortwave radiation, which does not vary as much from year to year, is dominant in the net heat flux during the summer season, the error in *Qu* is mostly due to the uncertainty in the sea ice concentration dataset. Previous studies have reported that derived ice concentrations are less reliable over melting and ponded sea ice^28^. Also, melt ponds act as a conduit for heat input into the upper ocean. Hence, calculations of *Qu* and its error need to consider the pond coverage of sea ice. Regarding the treatment of melt ponds in the bootstrap algorithm, we assume two extreme, bounding cases. Namely, all melt ponds are classified as either part of the sea ice or the open water fraction (Supplementary Fig. S8), providing estimates of the lower and upper bounds of *Qu* as the sum of heat absorbed through the open water fraction and transmitted from melt ponds. Note that the transmittance of melt ponds *τ* strongly depends on the ice thickness. Following previous studies which estimate the typical *τ* based on *in situ* observations, we use the value of 0.55 for the first-year ice^59^ and 0.17 for the multiyear ice^60^. The obtained *Qu* of the lower and upper bounds are shown by red shadings in Fig. 2.

The volume of ice melt is calculated as the product of ice area loss and mean ice thickness. The uncertainty of change in ice area obtained from the ice concentration is much smaller than that of ice thickness, for which much fewer data are available. In this paper, we assume that the uncertainty of ice melt is controlled by the uncertainty in mean ice thickness which is evaluated from the assumed initial ice thickness. From the 6-year observations by ICESat (Supplementary Fig. S3), mean ice thickness in the spring season exhibits a standard deviation of ±0.1 m relative to the average of 1.4 m. In this paper, we regard 2σ (±0.2 m) as the uncertainty, as indicated by gray shading in Fig. 2.

The uncertainty in estimating *Div* is due to errors in ice drift velocity and ice concentration. Based on error propagation, an uncertainty of *Div* can be represented as

18 $$\frac{{\delta }_{Div}}{\bar{Div}}=2\sqrt{2}\frac{{\delta }_{u}}{\bar{u}}+\frac{{\delta }_{C}}{\bar{C}},$$

where $\overline{Div}$ is the mean ice divergence, $\bar{u}$ is the mean ice velocity, $\overline{C}$ is the mean ice concentration, *δ*_*Div*_, *δ*_*u*_, and *δ*_*C*_ are errors in *Div*, *u*, and *C*, respectively. Here we assume *δ*_*u*_ ≈ 0.02 m s^−1^, based on documentation provided by the dataset originators^48^. In the early melt season, the analysis area is mostly covered by sea ice with *δ*_*C*_ of only a few percent; with a typical value of $\bar{u}$ being ~0.2 m s^−1^, the relative error ${\delta }_{C}/\overline{C}$ is hence one order of magnitude smaller than ${\delta }_{u}/\bar{u}$. Consequently, uncertainty in *Div* mainly originates from that of ice drift velocity and equation (18) can be reduced to ${\delta }_{Div}/\overline{Div}$ ≈ $2\sqrt{2}{\delta }_{u}/\bar{u}$. This uncertainty of *Div*, $2\sqrt{2}{\delta }_{u}/\bar{u}$ ≈ 28%, is shown by blue shadings in Fig. 2d.

## Electronic supplementary material

Supplementary Figures
