# Supplementary material for: Evidence for ice-ocean albedo feedback in the Arctic Ocean shifting to a seasonal ice zone
Source: Sci Rep. 2017 Aug 15;7:8170. doi: 10.1038/s41598-017-08467-z (PMC5557900; doi:10.1038/s41598-017-08467-z)
Supplement: Supplementary file 1 — Supplementary Figures [file 41598_2017_8467_MOESM1_ESM.pdf]

Supplementary Information

Observational evidences for ice-ocean albedo feedback in the Arctic Ocean shifting to seasonal ice zone

Authors: Haruhiko Kashiwase, Kay I. Ohshima, Sohey Nihashi, and Hajo Eicken

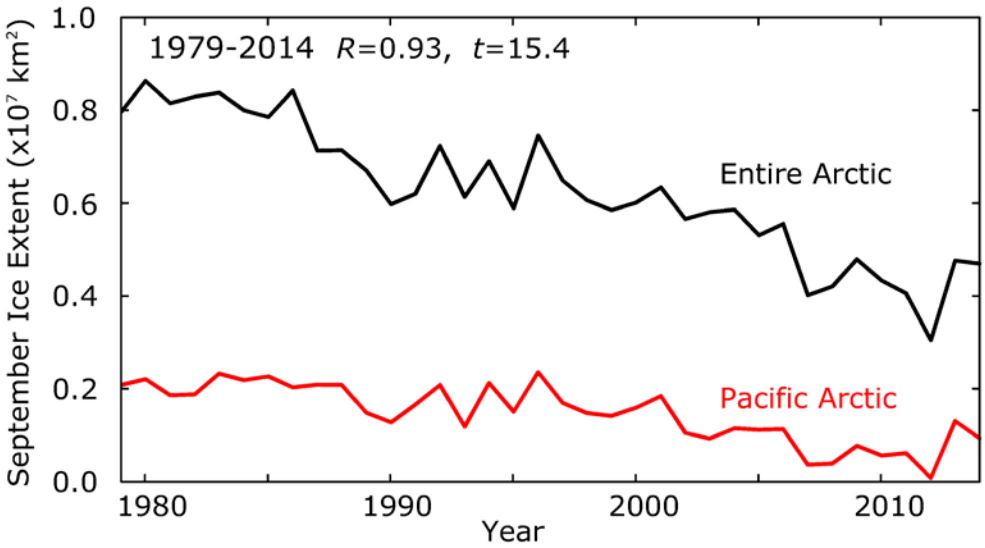

Figure S1 | Interannual variation of September sea ice extent. Black and red lines indicate the ice extent in the entire Arctic Ocean (north of the 65 °N) and the Pacific Arctic Sector (fan-shaped area in Figure 1), respectively. The ice extent is determined as the sum of grid cell area with ice concentration ≥15 %.

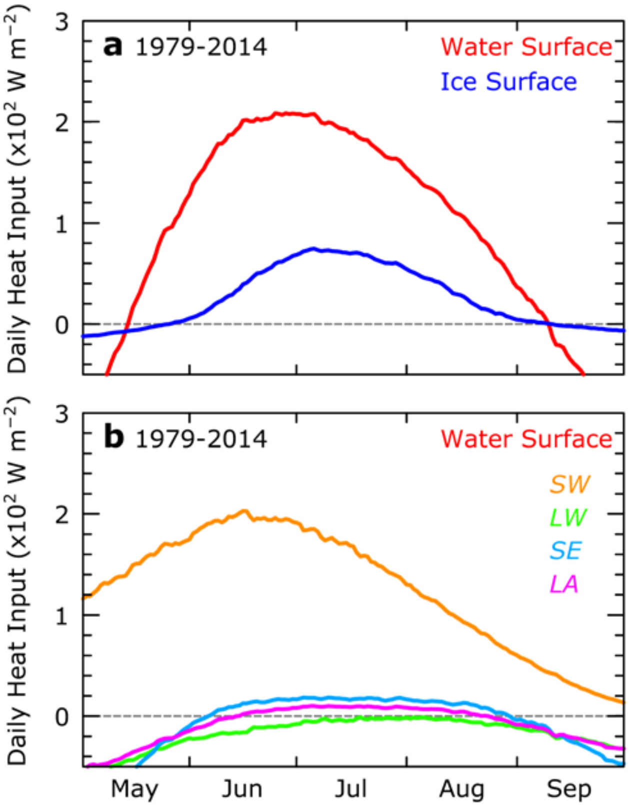

Figure S2 | Seasonal evolution of heat fluxes averaged over the period 1979–2014. (a) Net heat input at the water and ice surfaces (red and blue lines, respectively). (b) Individual terms in the heat budget at the water surface. *SW*, *LW*, *SE*, and *LA* indicate shortwave radiation, net longwave radiation, sensible heat flux and latent heat flux, respectively.

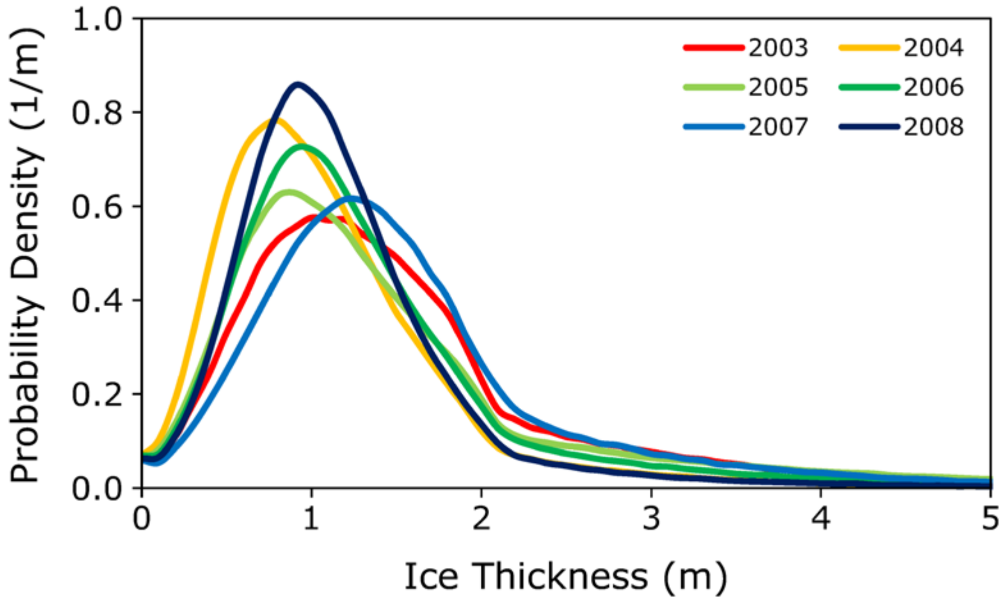

Figure S3 | Ice thickness distribution derived from ICESat observations. Probability density functions of ice thickness in the analysis area just before the onset of the melt season (February through May) are shown.

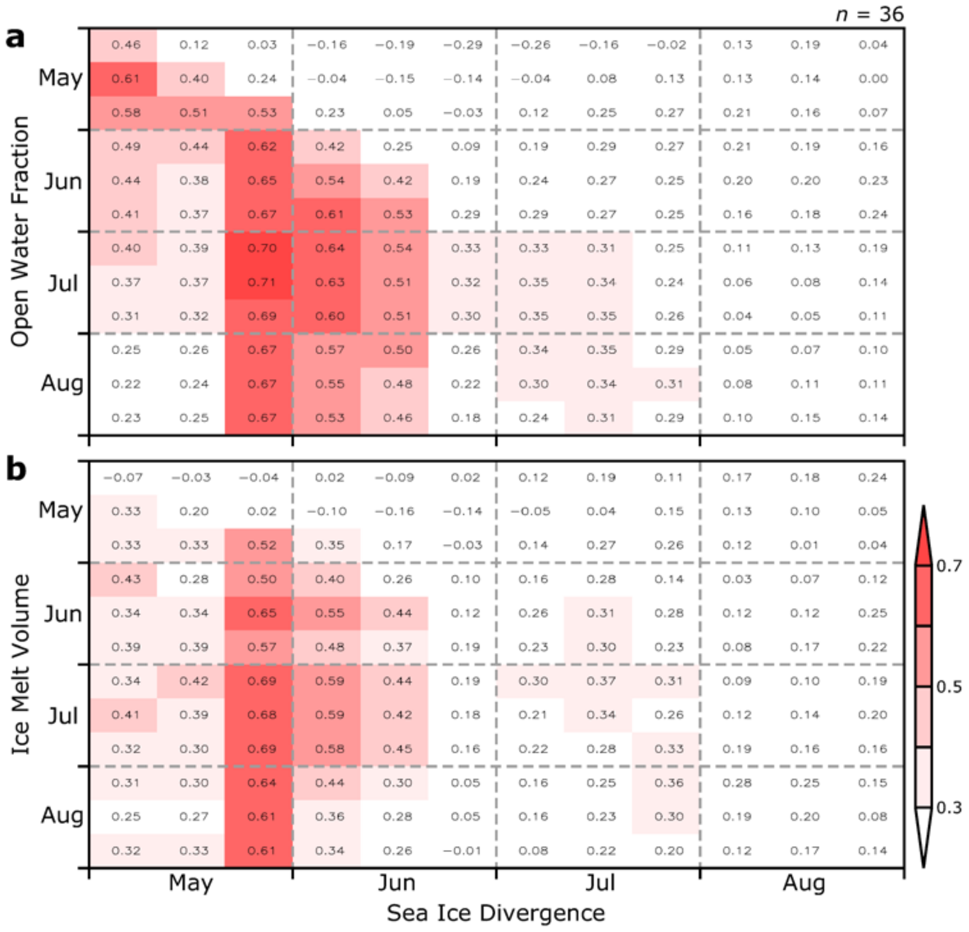

Figure S4 | Summary of lagged-correlation analysis. Correlation coefficients between the ice divergence and (a) open water fraction and (b) ice melt volume over the analysis area are shown. Note that all data are smoothed with one month running mean filter. A correlation coefficient ≥0.44 is statistically significant at a ≥99 % confidence level (n = 36).

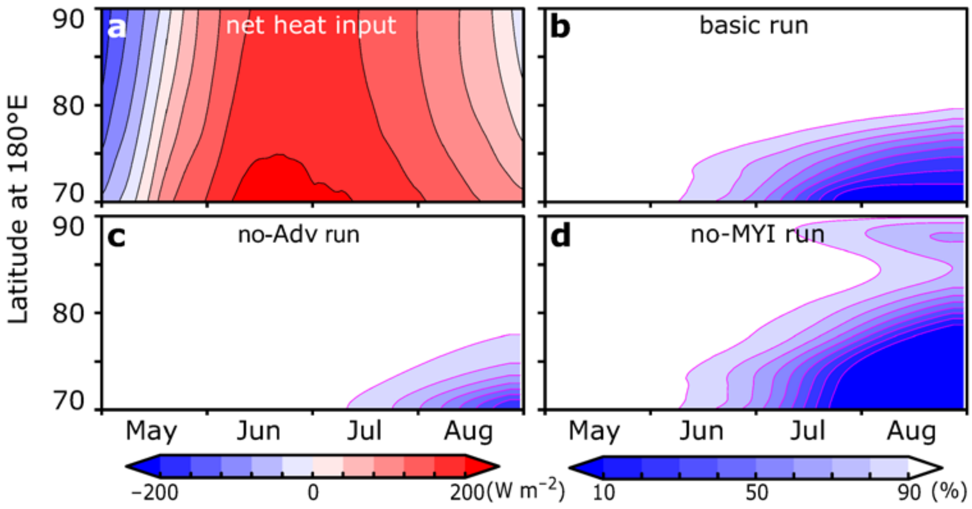

Figure S5 | Results from the simplified ice-ocean coupled model. Time evolution of (a) net heat input at the water surface obtained from the heat budget calculation and ice concentration obtained from (b) the basic run and experiments without (c) ice motion and (d) multiyear ice are shown.

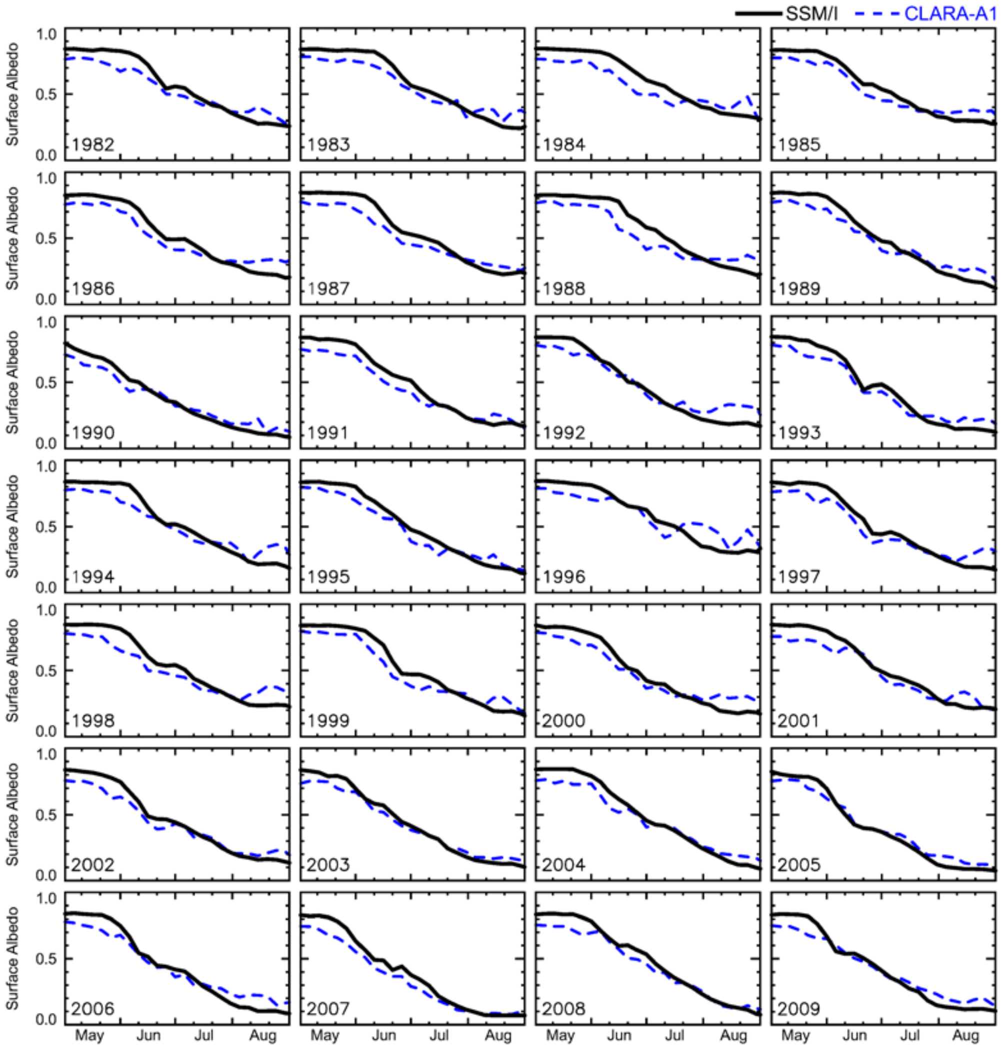

Figure S6 | Seasonal evolution of mean surface albedo averaged over the analysis area. The black solid line indicates albedo estimated from the empirical approach based on SSM/I observations. The blue dashed line indicates albedo directly observed by AVHRR (CLARA-A1[54]).

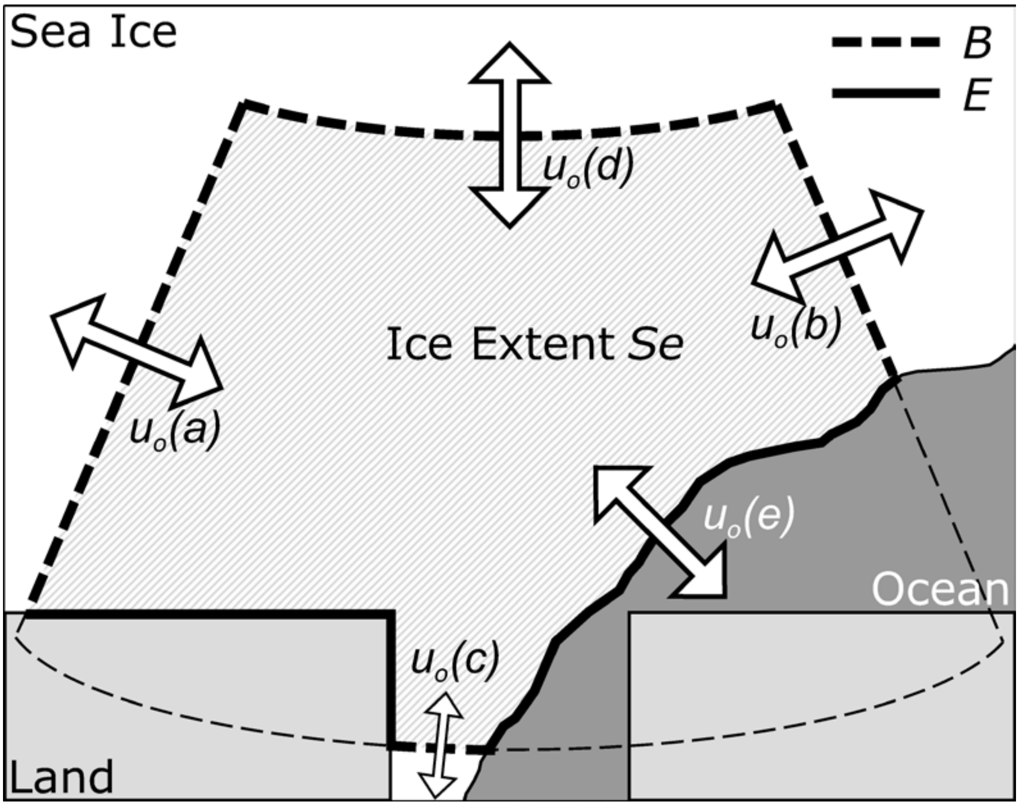

Figure S7 | Schematic diagram showing calculation of sea ice divergence. The dashed line indicates the boundary of the domain (*B*). The thick solid line marks the ice edge (*E*).

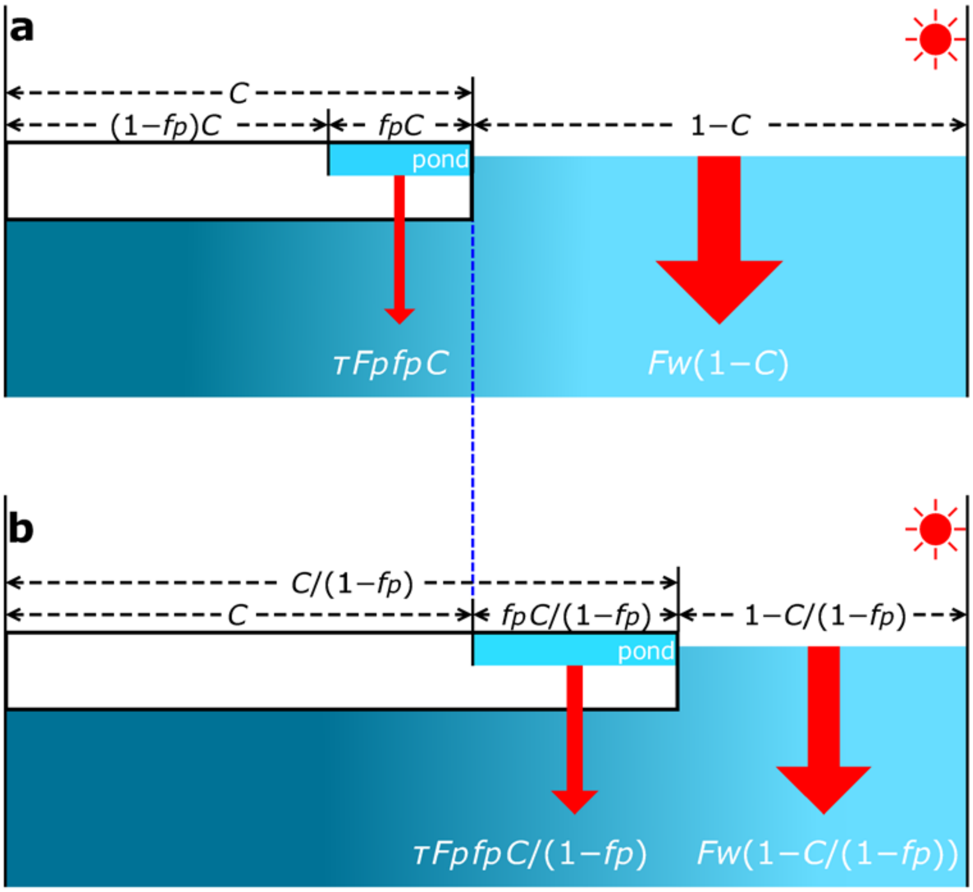

Figure S8 | Schematic diagram of the calculated heat budget. Two extreme cases of uncertainties associated with the melt pond fraction, i.e., cases when all ponds are classified as (a) sea ice area or as (b) open water fraction are shown. *C* is the ice concentration derived from SSM/I observations with the bootstrap algorithm, *fp* is the melt pond fraction, τ is the heat transmittance for melt ponds, and *Fp* and *Fw* are the net heat input per unit area at the pond and water surfaces, respectively.
